# A Novel Endoscopic Strategy for Addressing Complex Gastrointestinal Defects via the X‐Tack System: A Case Series With Videos

**DOI:** 10.1002/deo2.70251

**Published:** 2025-11-22

**Authors:** Robert Di Mitri, Giulio Calabrese, Filippo Mocciaro, Sandro Sferrazza, Elisabetta Conte, Anna Calì, Daniela Scimeca, Michele Amata

**Affiliations:** ^1^ Gastroenterology and Endoscopy Unit ARNAS Civico ‐ Di Cristina – Benfratelli Palermo Italy; ^2^ Clinical Medicine and Surgery University of Naples Federico II Naples Italy

**Keywords:** endoscopic closure, endoscopic suturing, gastrointestinal defect, post‐surgical complication, through‐the‐scope closure

## Abstract

The management of complex gastrointestinal defects (CGDs), such as fistulas, leaks, and anastomotic dehiscence, remains challenging. Over‐the‐scope suturing (OTSS) systems provide effective closure, but their application is limited to specific anatomical sites. The X‐Tack through‐the‐scope suturing (TTSS) system offers a minimally invasive alternative for CGD closure without requiring scope withdrawal. This case series evaluated patients who underwent TTSS closure for CGDs ≤25 mm at our center. All patients were assessed through computed tomography and multidisciplinary board discussion before endoscopic treatment. Endoscopic closure was performed using the X‐Tack system, applying a figure‐of‐8 or zig‐zag pattern depending on defect characteristics. Follow‐up included clinical and laboratory assessments at seven days and during long‐term observation. Thirteen patients underwent TTSS closure, achieving a 100% technical success rate. Clinical and laboratory remission at seven days was observed in 76.9% of cases, with a sustained clinical remission in 76.9% after a median follow‐up of 2.5 months. The procedure was effective even in challenging anatomical locations, including post‐surgical anastomotic leaks and narrow lumens. TTSS using the X‐Tack system is a safe and effective approach for small CGD closure, particularly in difficult anatomical sites.

## 1 Introduction

Advancements in endoscopic techniques for complex gastrointestinal defect (CGD) closure have equipped endoscopists to effectively address leaks, fistulas, perforations, and a range of mucosal defects [1]. Over‐the‐scope suturing (OTSS) systems allow for an effective and safe full‐thickness soft tissue approximation [2]. However, the need for scope withdrawal and its limited maneuverability reduces the possibility of performing closure on specific sites, like the duodenum, right colon, or narrowed anastomoses.

To overcome this limitation, a through‐the‐scope suturing (TTSS), called the X‐Tack device (Boston Scientific), was developed in 2020. The X‐Tack device can be used in ≥ 2.8 mm channel endoscopes and enables the positioning of the closing helix to approach mucosal margins to protect extraluminal tissue. X‐Tack has been proven effective and safe for different types of defects, starting from post‐resection to post‐peroral endoscopic myotomy (post‐POEM) defect closure [3].

Recently, Canakis et al. [4] have reported a multicenter retrospective experience regarding the endoscopic closure of different types of gastrointestinal (GI) defects. Although the TTSS system was performed in a complete fashion for post‐resection closures, it did not achieve clinical efficacy for fistula, leaks, and perforation repairs.

Based on these assumptions, we present a case series assessing the effectiveness and safety of TTSS systems for closing CGDs, including fistulas, leaks, and anastomotic dehiscence.

## 2 Procedure and Technique

We conducted a single‐center retrospective observational study in an Italian Tertiary Referral Centre. We included all consecutive patients undergoing endoscopic suturing closure with TTSS systems for CGDs, such as fistula, leaks, and dehiscence, with a maximum diameter of 25 mm, between March and September 2024. During the study period, all post‐surgical GI defects ≤25 mm were treated using TTSS. No other closure systems were used.

**FIGURE 1:**
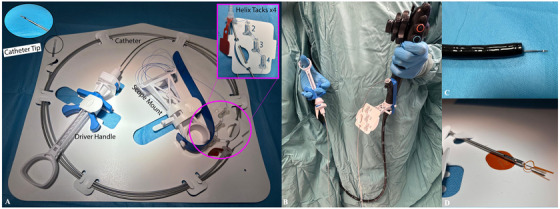
Illustration of the X‐Tack system components. (A) Labelled layout of the X‐Tack Endoscopic HeliX Tacking System, showing the catheter, driver handle, and scope mount. (B) Clinical setup of the X‐Tack device mounted on the endoscope, with the driver handle in the assistant's hand. (C) Endoscopic catheter tip with preloaded tack visible at the distal end of the scope, positioned for precise delivery to the target site. (D) Close‐up view of the cinch loading tool.

The X‐Tack TTSS system (Boston Scientific, MA, USA), illustrated in Figure 1, is delivered through the endoscope's working channel (minimum 2.8 mm) without requiring scope withdrawal. Once the catheter is in position, helical tacks are sequentially deployed along the margins of the defect. After placement, a cinching device is advanced to tension the suture, approximating the mucosal edges and achieving closure. The suture is then locked and cut to complete the procedure [5].

### 2.1 Included Patients

We collected data regarding age, gender, type of GI defect, previous surgical procedures, time between procedure and endoscopic closure, and previous closure treatment. We also collected data regarding the site, CGD type, and the status of the surrounding mucosa.

### 2.2 Outcomes and Follow‐up

The primary outcome of the study was technical success, defined as the complete endoscopic closure of the GI defect using the TTSS system without the need for additional endoscopic or surgical intervention during the procedure.

Secondary outcomes were evaluated as follows:

‐ clinical remission at seven days, defined as resolution of presenting symptoms;
‐ laboratory remission, defined as normalization of inflammatory markers;
‐ laboratory response, defined as a ≥30% reduction in leukocyte count, C‐reactive protein (CRP), and procalcitonin (PCT);
‐ sustained clinical remission, defined as continued symptom resolution without recurrence at follow‐up.

Laboratory response cut‐off was pragmatically chosen as an exploratory outcome to reflect early biological improvement, in line with prior studies using serial inflammatory markers to detect postoperative complications, despite the lack of a standardized threshold.

Moreover, depending on each patient's available observational time, disease recurrence and complications were registered during different follow‐up periods.

## 3 Defect Description and Endoscopic Management

At baseline, all patients reported symptoms/signs (dysphagia, vomiting, abdominal pain, perianal pain, fever, sepsis) or laboratory alterations (increase in leukocytes, CRP, PCT) consistent with GI wall defects. All of them were evaluated with a computed tomography (CT) scan plus an oral gastrografin radiography when indicated for dynamic evaluation. A multidisciplinary board discussion was conducted among surgeons, radiologists, and gastroenterologists to determine the best therapeutic approach. The endoscopic closure technique was chosen to minimize the risk of possible surgery‐related complications.

All procedures were performed by a single endoscopist with extensive experience in advanced endoscopy, including therapeutic endoscopic retrograde cholangiopancreatography and endoscopic ultrasound, in a hybrid operating room that allowed for fluoroscopy to dynamically assess the nature of the defect and ascertain closure when needed. Before clinical use of the TTSS system, the operator had performed over 20 endoscopic suturing procedures with over‐the‐scope devices and completed dedicated hands‐on training with the X‐Tack system on ex vivo and in vivo animal models under the supervision of manufacturer‐appointed specialists.

Procedures were conducted with the anesthesiologist's assistance. General anesthesia was performed for upper GI cases.

After providing written informed consent, all patients underwent closure with the TTSS system “X‐Tack” (Boston Scientific, MA, USA). Two different tool lengths (160 or 230 cm) were applied, and the number of “tacks” used varied according to the defect size. Depending on the shape of the defect and the scope angle, a figure‐of‐8 or zig‐zag pattern was applied to each closure. Although an optimal configuration has not been formally established, we followed available literature [6] by using a zig‐zag pattern for linear and crescent‐shaped defects, and a figure‐of‐8 configuration for circular lesions (with tacks placed diagonally in a criss‐cross manner across the defect).

TTSS was preferred over OTS clips for its better maneuverability in narrow spaces and deployment without scope withdrawal, useful in surgically altered anatomy.

Depending on the site of the defect, the procedure was performed with standard or therapeutic gastroscopes (Fujifilm 760R/760CT) or colonoscopy (Fujifilm 760R‐V/M). Just in one case, a double‐balloon enteroscope (DBE) (Fujifilm 580BT) was preferred over medium—and long‐length standard colonoscopes due to its increased stability and maneuverability. The goal was to reach and treat a jejunal leak after Roux‐en‐Y duodeno‐jejunostomy (RYDJ).

All patients who showed fluid collections received systemic antibiotic therapy, and radiologic follow‐up confirmed reabsorption of the collections, even when clinical improvement had already been achieved, to ensure adequate cavity reduction before closure. No cases required additional drainage, as the collections were small and self‐limiting.

## 4 Results

### 4.1 Included Population

Thirteen patients received endoscopic closure of CGDs with the TTSS system: 10 (76.9%) males and three (23.1%) females. The median age was 57 (interquartile range: 48–67). The median American Society of Anesthesiologists scores and the Charlson Comorbidity Index (CCI) were 3 [2, 3] and 5 [3, 4, 5], respectively. CGDs belonged to the esophagus in four cases, to the duodenum in one, to the jejunum in two, and to the colon and the rectum in three each.

The type of surgery or endoscopic procedure previously performed was total/partial gastrectomy in four cases (30.8%), ileal or colonic resection in four cases (30.8%), two cases of biliopancreatic surgery (15.4%), one case of POEM (7.7%), and two cases of endoscopic submucosal dissection (15.4%). The time between the procedure and the endoscopic closure widely varied between 3 and 210 days.

Five (38.5%) cases had previously undergone endoscopic treatment: in two cases, TTS clips were positioned, and in the other two, a self‐expandable metal stent (SEMS) was used. Moreover, several endoscopic attempts were made through SEMS and VAC‐Stents in one case. Table 1 summarizes the included cohort's clinical characteristics.

**TABLE 1:** Overview of patients’ data, procedure, and clinical outcomes, case‐by‐case reported in chronological order.

	Case 1	Case 2	Case 3	Case 4	Case 5	Case 6	Case 7	Case 8	Case 9	Case 10	Case 11	Case 12	Case 13
Age (y)	67	61	48	52	56	76	75	26	41	41	88	51	58
Gender	F	F	M	M	M	M	M	M	M	M	M	M	F
Diagnosis	Duodenal leak	Colonic fistula	Ileo‐colonic anastomotic dehiscence^*^	Rectal perforation	Jejunal leak	Colo‐rectal anastomotic dehiscence	Esophago‐jejunal anastomotic dehiscence	Esophago‐pleural fistula	Colo‐colonic anastomotic dehiscence	Esophageal covered perforation	Colo‐rectal anastomotic dehiscence	Esophago‐jejunal anastomotic dehiscence	Esophago‐jejunal anastomotic dehiscence
Defect site	Duodenum	Descending colon	Colon	Rectum	Jejunum	Rectum	Jejunum	Esophagus	Transverse colon	Esophagus	Rectum	Esophagus	Esophagus
Defect width (mm)	8	10	25	3	20	5	10	7	15	20	10	20	20
Presence of fluid collection	No	Yes	Yes	Yes	No	No	No	Yes	No	No	No	No	Yes
Healthy wall condition	Yes	Yes	No	No	No	Yes	No	No	Yes	No	Yes	Yes	No
Type of procedure	B‐P surgery	Total gastrectomy	Ileo‐colonic resection^*^	ESD	Roux‐en‐Y duodeno‐jejunostomy	Anterior rectal resection	Partial gastrectomy	POEM	Colonic resection	ESD	Anterior rectal resection	Total gastrectomy	Total gastrectomy
Time from surgery/index event	14 d	10 w	8 m	7 d	14 d	36 d	1 m	7 m	6 m	3 d	30 d	15 d	18 d
Previous endoscopic treatment	None	Multiple attempts	Clip placement	Clip placement	None	None	None	SEMs placement	None	None	None	None	SEMs placement
General anesthesia	Yes	Yes	Yes	No	Yes	No	Yes	Yes	No	Yes	No	Yes	Yes
Type of scope	TG	TG	SC	TG	DBE	TG	TG	TG	TG	SC	TG	TG	TG
Number of “tacks” placed	4	8	12	4	6	4	4	4	8	8	4	12	16
7‐day clinical remission	Yes	No	Yes	Yes	No	Yes	Yes	Yes	Yes	Yes	Yes	No	Yes
7‐day laboratory response	Yes	No	Yes	Yes	Yes	Yes	Yes	Yes	Yes	Yes	Yes	Yes	Yes
Long‐term remission	**Yes**	No	**Yes**	**Yes**	**Yes**	**Yes**	**Yes**	No (fistula recurrence)	**Yes**	**Yes**	**Yes**	No	**Yes**

Abbreviations: B‐P, biliopancreatic; DBE, double balloon enteroscope; ESD, endoscopic submucosal dissection; F, female; M, male; POEM, peroral endoscopic myotomy; SC, standard colonscope; SEMs, self‐expandable metal stent; TG, therapeutic gastroscope.

* Ileo‐colonic anastomotic dehiscence in Crohn's disease.

### 4.2 Procedure and Main Outcomes

Endoscopic closure using the X‐tack system was completely achieved in all the cases, with 100% technical success.

The median closure time was 25 (18–40) min, with a median number of 6 [4, 5, 6, 7, 8] “tacks” positioned; more than four “tacks” were needed in seven (53.8%) out of 13 cases.

The median width of the defect was 10 (8–20.0) mm, and nine (69.2%) out of 13 cases presented unhealthy conditions, defined as diffusely edematous and pale mucosa surrounding the wall defect.

After 7 days, 10 (76.9%) patients achieved clinical remission and laboratory response, two (15.4%, cases 5 and 12) achieved only laboratory response, and one (7.7%, case 2) did not gain any benefit. The latter three cases repeated a CT scan, confirming the persistence of a GI defect in just two cases (cases 2 and 12). Case 5 had achieved a laboratory response and progressively a complete reduction of the output from the abdominal drains and so confirming its resolution as secondary intention healing (Video S1). Drains were removed after 14 days, thanks to the total improvement of clinical conditions. Case 12, which presented at the beginning a severe, unhealthy condition of the mucosa with edema and irregular borders, received a novel treatment with a fully‐covered SEMS with an anti‐migration system (Beta‐2 stent, Taewong), achieving no benefit during the follow‐up and consequently undergoing a new surgical intervention. Regarding case 2, the patient did not undergo additional treatment due to compromised systemic conditions. Notably, in addition to the colonic fistula, this patient had an esophageal‐jejunal leak and an abdominal abscess developed after gastrectomy. After one month, the patient died.

Concerning the long‐term remission, 10 achieved a sustained clinical and laboratory remission after a median follow‐up of 2.5 (1.8–4.5) months. While another patient (case 8) developed a new fistula recurrence and was scheduled for a surgical esophagectomy intervention, although the CT‐scan reported an adequate reduction of the thoracic collection and a correct closure of the GI defect. In conclusion, our long‐term clinical success was 76.9 % (10/13 patients).

**FIGURE 2:**
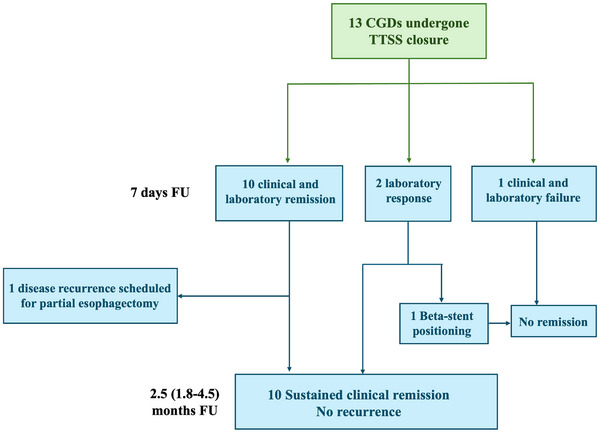
Flowchart of main outcomes.

Figure 2 and Table 2 report the procedures and the main outcomes of the cohort.

**TABLE 2:** Summary of procedural and clinical outcomes of the case series.

	Patients (*n* = 13)
Complete closure, *n* (%)	13 (100%)
Median procedure time, min (IQR)	25 (18–40)
Median defect width, mm (IQR)	10 (8‐20.0)
7‐day clinical remission, *n* (%)	10 (76.9%)
Sustained clinical remission, *n* (%)	10 (76.9%)
Median follow‐up, months (IQR)	2.5 (1.8–4.5)

Abbreviations: *n*, number; IQR, interquartile range.

## 5 Discussion

Endoscopic management of CGDs remains challenging, and specific procedural guidelines are lacking. TTSS systems have been part of the endoscopists’ armamentarium since 2020 [7]. However, they are not designed for full‐thickness closure, and their effectiveness in thick‐wall organs (stomach, ileocecal valve, rectum, colon, and esophagus) is limited by suture tension. Consistently, in these sites, its main function has traditionally been considered for mucosal apposition after endoscopic resections or POEMs [4, 8]. However, in some cases where the defect is positioned at an acute angle, with difficulty in achieving good exposure, a through‐the‐scope device can become helpful, being maneuverable and not suffering from the scope bending when the “tacks” must be released. We used TTSS for <25 mm defects in challenging sites, achieving 100% technical and 76.9% clinical success. The operator performing closure was a highly experienced endoscopist, but did not have previous experience in X‐Tack positioning on human cases before the enrollment started. The successful completion of all procedures without prior system‐specific training suggests that the device is user‐friendly and may require only a limited familiarization period for experienced operators [7].

Moreover, a through‐the‐scope device allows the endoscopist to reach technically difficult regions without the necessity to withdraw the scope, such as a Roux‐en‐Y altered anatomy (Case 5—Video S1) or a blind loop duodenum after surgical gastroentero‐anastomosis (Case 1—Video S2), reaching a jejunal and duodenal leak, respectively.

Specifically, case 5 was performed using a DBE, allowing the endoscopist to access the hepato‐jejunal anastomosis after multiple failed attempts with colonoscopes.

At the same time, despite being easily reachable, working in narrow lumens, like in the esophagus in case 10, can become challenging, especially with mounted devices like OTS systems.

Using OTSS systems for closure, like Overstitch or OTS clips, for cases like the ones described above would have, indeed, been difficult and limited by the acute angles encountered by the scope. The risk of incorrectly releasing the OTS clip could seriously affect the outcome of a good procedure and hinder other possible rescue treatments.

Moreover, the use of OTS clips on unhealthy and friable mucosal flaps should be reduced in order to avoid the risk of tissue disruption and prevent further complications [9]. In our cohort, 53.8% (7/13) of cases were performed in edematous and friable wall conditions, which were avoided by the operator during the placement of each “tack” in order to include it in the closure and prevent tissue disruption.

In addition to technical feasibility, the durability of closure is a critical factor, particularly in CGDs involving fibrotic or indurated tissue, where healing is often delayed and the mechanical tension on the suture remains high over time. In our series, no delayed clinical failures were observed during follow‐up, suggesting that stable closure can be achieved in selected cases, although longer‐term and larger‐scale data are needed to confirm these outcomes.

In the recent multicenter experience reported by Canakis et al. [4], the effectiveness of the TTSS systems was limited in treating fistula, leaks, and perforation. Specifically, they report a 54.5% and 28.6% clinical benefit for fistula and leaks. Even though it was not possible to extract specific data on the median diameter of the defects included, we can assume from the general data that they were larger in size than ours. This factor may have influenced the rate of clinical success of their cohort. Similarly, in a retrospective experience conducted by Krishnan et al. on a median defect size of 32.6 mm [10], the rate of clinical success was 57.1%. Notably, clinical success rates with OTSC systems have also been shown to be significantly lower in the treatment of fistulas, particularly due to the presence of chronic fibrotic tissue and poor tissue compliance, which limit adequate capture of the defect. Prior studies have demonstrated that OTSC closure is most effective in fistulas smaller than 10 mm, suggesting that defect size is a key determinant of success across closure systems, including TTSS13.

Even though our experience is limited to 13 patients from a single center, selecting <25 mm in‐size defects may have allowed us to achieve higher clinical success. Indeed, when the operator notes that closure may be insufficient and the defect may be too large, an additional treatment could be performed with adjunctive devices. A recent meta‐analysis [11] highlighted how the adjunctive role of TTS clips can optimize the closure's feasibility. In fact, the defect closure rate increased from 77.2% to 95.2%.

Finally, we report using this TTSS system for small anastomotic dehiscence, highlighting its effectiveness even on different tissue types, like the esophagus, colon, and rectum. One case belongs to a Crohn's disease patient. To date, this is the first experience reporting the feasibility of TTSS closure of small CGDs, focusing on post‐surgical fistula, leaks, or dehiscence.

A multi‐center prospective study is awaited to position TTSS in the closure of GI defects and determine the ideal size of the defect to consider, especially when full‐thickness closure (like in fistulas and leaks) is needed.

## 6 Conclusions

In conclusion, this single‐center case series suggests that TTSS may be a safe and technically feasible option for closing small post‐surgical GI defects, especially in challenging anatomical sites. The presence of unhealthy mucosa surrounding the defect does not limit its feasibility. On the contrary, large defects may not be an ideal candidate for this kind of closure. However, larger comparative and prospective studies are needed to better define its role and long‐term outcomes.

## Author Contributions

All authors gave substantial contributions and approved the final version of the manuscript. **Di Mitri Roberto**: conceptualization, supervision, and writing—review & editing. **Calabrese Giulio**: data curation, formal analysis, and writing—original draft preparation. **Mocciaro Filippo**: writing—review & editing. **Scimeca Daniela**: writing—review & editing. **Calì Anna**: writing—review & editing. **Conte Elisabetta**: writing—review & editing. **Sferrazza Sandro**: writing—review & editing. **Amata Michele**: writing—original draft preparation and methodology.

## Conflicts of Interest

The authors declare no conflicts of interest.

## Funding

The authors received no specific funding for this work.

## Ethics Statement

This study was approved by the Ethics Committee Palermo 2 (Approval ID: 10/02). The study was conducted in accordance with the Declaration of Helsinki.

## Consent

Obtained from individuals.

## Clinical Trial Registration

N/A

## Supporting information




**VIDEO S1** Hepato‐jejunal anastomotic leak after left hepatectomy with Roux‐en‐Y reconstruction successfully treated with X‐Tack system using a short double balloon enteroscope.

**VIDEO S2** Mini‐invasive endoscopic approach for a post‐cholecystectomy duodenal leak: retrograde access into the duodenal blind loop and endoscopic suturing using the X‐Tack device.
